# Sequencing SARS-CoV-2 from antigen tests

**DOI:** 10.1371/journal.pone.0263794

**Published:** 2022-02-08

**Authors:** Ashley Nazario-Toole, Holly M. Nguyen, Hui Xia, Dianne N. Frankel, John W. Kieffer, Thomas F. Gibbons

**Affiliations:** 1 59th Medical Wing, Clinical Investigations and Research Support Laboratory, Joint Base San Antonio-Lackland, San Antonio, TX, United States of America; 2 Trainee Health Surveillance, 559th Medical Group, THLS, Joint Base San Antonio-Lackland, San Antonio, TX, United States of America; University of Helsinki: Helsingin Yliopisto, FINLAND

## Abstract

Genomic surveillance empowers agile responses to SARS-CoV-2 by enabling scientists and public health analysts to issue recommendations aimed at slowing transmission, prioritizing contact tracing, and building a robust genomic sequencing surveillance strategy. Since the start of the pandemic, real time RT-PCR diagnostic testing from upper respiratory specimens, such as nasopharyngeal (NP) swabs, has been the standard. Moreover, respiratory samples in viral transport media are the ideal specimen for SARS-CoV-2 whole-genome sequencing (WGS). In early 2021, many clinicians transitioned to antigen-based SARS-CoV-2 detection tests, which use anterior nasal swabs for SARS-CoV-2 antigen detection. Despite this shift in testing methods, the need for whole-genome sequence surveillance remains. Thus, we developed a workflow for whole-genome sequencing with antigen test-derived swabs as an input rather than nasopharyngeal swabs. In this study, we use excess clinical specimens processed using the BinaxNOW™ COVID-19 Ag Card. We demonstrate that whole-genome sequencing from antigen tests is feasible and yields similar results from RT-PCR-based assays utilizing a swab in viral transport media.

## Introduction

As of January 2022, severe acute respiratory syndrome coronavirus 2 (SARS‐CoV‐2) has resulted in over 5.5 million deaths worldwide, with more than 836,000 deaths in the United States of America alone [1]. Due to the continuing evolution of the pandemic, ongoing genomic surveillance of SARS-CoV-2 is critical for identifying emerging variants [2–4], confirming cases of reinfection [5], and vaccine breakthrough cases [6], all of which provide information to be considered when implementing any changes to public health policies [7].

To assist Department of Defense (DoD) public health officials involved in tracking the evolution of SARS‐CoV‐2 variants, the 59^th^ Medical Wing (59^th^ MDW) Clinical Investigations Research Support (CIRS) Sequencing & Bioinformatics Laboratory spearheaded efforts to sequence the virus from excess clinical specimens collected from Joint Base San Antonio (JBSA) DoD beneficiaries. Specimens sequenced from April 2020 to April 2021 were Nasopharyngeal (NP) swabs that had been previously tested for SARS-CoV-2 by the Wilford Hall Ambulatory and Surgical Center Clinical laboratory. When properly stored, residual NP specimens produce high-quality, SARS-CoV-2 whole genome sequencing (WGS) data [8–10]; as demonstrated by the fact that NP swabs in VTM/PBS are the most widely accepted sample source for most SARS-CoV-2 WGS pipelines [11, 12]. In May 2020, the Food and Drug Administration granted its first emergency use authorizations (EUAs) to lateral flow and immunoassay tests detecting SARS-CoV-2 protein antigens; including several over the counter, home antigen tests [13]. Since becoming available, antigen tests have been widely accepted as a suitable tool for global monitoring of new SARS-CoV-2 cases. In March 2021, the FDA issued an EUA for Abbott Diagnostic’s BinaxNOW™ COVID-19 Ag Card [13] and, concurrently, the DoD adopted antigen testing to screen asymptomatic active duty and DoD civilian personnel [14, 15].

Antigen tests differ from NP specimens collected for RT-PCR in several ways. First, antigen tests use anterior nasal (AN) swabs [16–18]. Additionally, NP swabs are stored in VTM, while antigen test swabs are inserted into an antigen card with no residual media available for additional testing [10, 17]. Given that our previously published methods relied on excess SARS-CoV-2 positive NP swabs in VTM, the lack of residual specimen from antigen cards presented a problem with respect to our ability to continue to conduct SARS-CoV-2 genomic surveillance at JBSA [19]. Thus, we developed a method to sequence SARS-CoV-2 from residual antigen test cards, thereby maximizing the options available to 59^th^ MDW Public Health Officials conducting COVID-19 surveillance. In this report, we demonstrate that it is possible to produce high quality genomic surveillance data from antigen-derived AN swabs. Specifically, we validated PCR-based whole genome sequencing from AN swabs utilized with the BinaxNOW™ COVID-19 Ag Card (Abbott Diagnostics Scarborough).

## Results

### Sample preparation optimization

**Fig 1:**
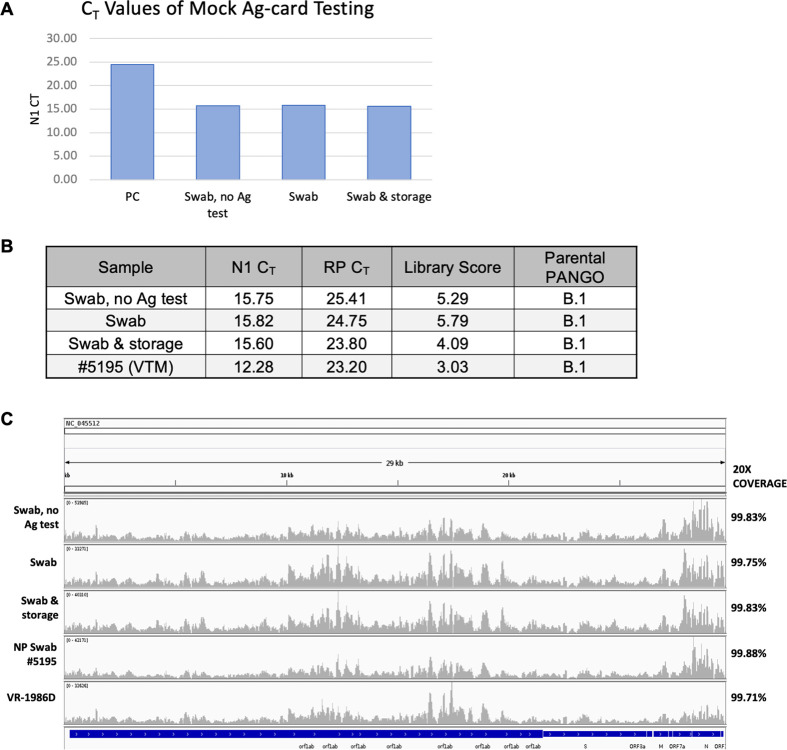
Assay development. (A) An NP specimen was used to evaluate the feasibility of obtaining viral RNA from Ag-card derived specimens. RT-PCR was carried out on a positive control and on mock Ag-card specimens collected under each condition. (B) RT-PCR N1 and RP Ct values, sequence library quality scores, and viral PANGO lineage assignments of antigen card specimens and reference NP specimen #5195. (C) IGV screen shots of sample SARS-CoV-2 genome coverage. VR-1986D = Positive Control Genomic RNA from SARS-Cov-2, Isolate USA-WA1/2020.

To optimize sample collection and preparation methods, we utilized a previously sequenced SARS-CoV-2 positive NP specimen with an N1 C_T_ of 12.28 (# 5195) (**Fig 1**). First, we used RT-PCR to examine the potential for sample loss and degradation due to (1) exposure to the BinaxNOW™ proprietary extraction buffer and (2) storage conditions (**Fig 1A**). In this, and every following RT-PCR experiment, we included a standardized positive extraction control, which is a COVID-19 positive NP specimen diluted in DNA/RNA Shield to N1 C_T_ ~25. We also included a water extraction and no template control. In Fig 1A, the positive control N1 C_T_ is shown; however, the water extraction and template controls are not shown, due to their expected and observed lack of amplification. To assess sample loss and degradation, a BinaxNOW™ COVID-19 Ag Card swab was dipped into NP specimen # 5195 and then placed into 500 μL DNA/RNA shield (*swab*, *no Ag test*). For comparison, a second swab dipped into NP specimen # 5195 was run through the antigen test for 15 min per manufacturer’s instructions and then placed into DNA/RNA shield (*swab*). The N1 C_T_ of the “swab, no Ag test” sample was 15.75 while the N1 C_T_ of the “swab” sample was 15.82, indicating that little to no viral RNA was lost after 15 min exposure to the Ag-card extraction buffer. To test if prolonged storage of antigen cards might affect sample yield, a third swab was dipped into the NP specimen, run through the antigen test, and the whole card was stored at 4°C for 2 hours before the swab was placed in DNA/RNA Shield (*swab & storage*). Again, RT-PCR results revealed that there was no sample degradation, as observed through N1 C_T_ values of 15.82 (*swab*) and 15.60 (*swab & storage*). PCR-amplicon sequencing libraries were then prepared for each sample using the Paragon Genomics CleanPlex Flex SARS-CoV-2 Panel (**Fig 1B & 1C**). For a library prep and sequencing control, we also prepared a library using commercially available SARS-CoV-2 genomic RNA (ATCC # VR-1986D, SARS-Cov-2 Isolate USA-WA1/2020). Library quality scores were calculated as previously described and samples were sequenced at 2x151 bp reads on the Illumina MiSeq system. N1 C_T_, library quality ratio scores, 20X genome coverage (20 or more reads mapped per nucleotide), and viral PANGO lineages are shown in **Fig 1B**. As expected, the positive control VR-1986D was assigned to PANGO lineage A and all dipped Ag-card specimens were assigned to PANGO lineage B.1, the same lineage assigned to NP specimen 5195. IGV snapshots show that all samples map at greater than 99.5% at 20X coverage across the genome, with over 99.8% coverage at 100X or greater & less than 0.02% of the genome covered by one or fewer bases (**Fig 1C**). Together, these finding indicate that sample exposure to Ag-card buffer and testing conditions does not impact the quality of RNA and subsequent sequencing steps.

### Performance comparison

**Fig 2:**
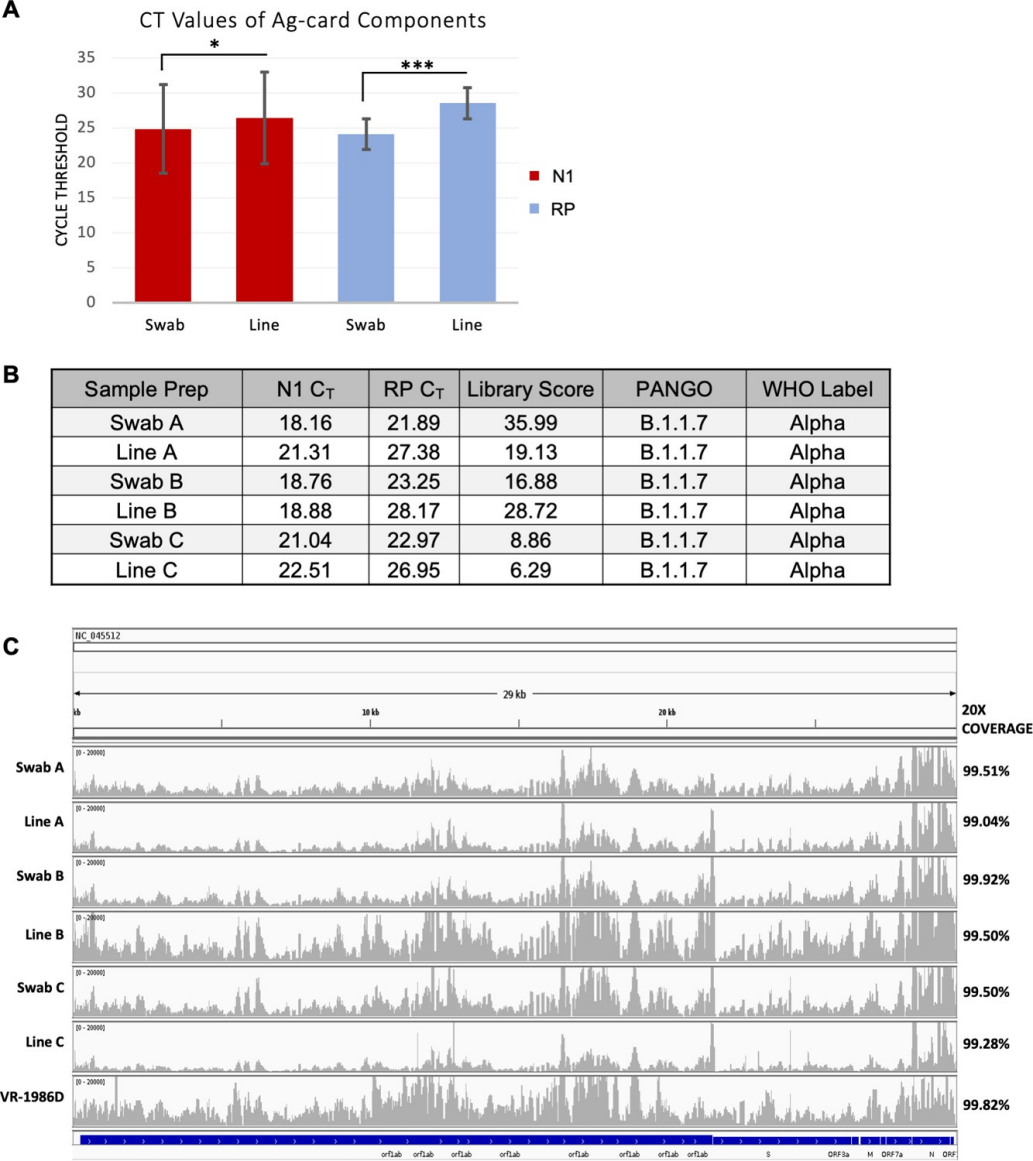
Sample source performance comparison. (A) SARS-CoV-2 positive BinaxNOW™ COVID-19 Ag Cards were used to evaluate which part of the card, swab or lateral flow positive line, yielded the highest quantity and quality of viral RNA. Extracted RNA was tested for N1 and RP levels using RT-PCR. The average N1 and RP C_T_ values are plotted. n = 7 cards. Statistical analysis = Two-tailed, paired *t-*test, * p<0.05; *** p<0.0005 (B) N1 and RP Ct values, library quality scores and PANGO lineage assignments. (C) IGV screen shots of SARS-CoV-2 genome coverage.

Given that we carried out our preliminary tests in a mock fashion by dipping swabs in a previously tested NP specimen, we next utilized COVID-19 positive Ag-cards to test the viability of sequencing from real specimens (excess clinical specimens collected under IRB-approved protocol FWH20200103E). Seven completed SARS-CoV-2 positive BinaxNOW™ COVID-19 Ag Cards were delivered to the lab and immediately processed as follows: Antigen cards were disassembled and the AN swabs and positive line of the lateral flow strips were separately placed into DNA/RNA shield, stored at 4°C for 48 hours, extracted, then amplified using RT-PCR (**Fig 2A**). We hypothesized that due to the nature of the lateral flow assay, viral RNA would be concentrated on the positive line of the strip. We found that swabs slightly outperformed the positive line by yielding higher viral loads in all samples. Specifically, swab N1 C_T_ values were, on average, 1.57 C_T_s lower than the line (± 1.16 C_T_s) (p-value = 0.0116). Interestingly, host RNA levels were significantly higher in the swabs: RP was detected in swab specimens 4.43 cycles sooner than the line (± 1.30 C_T_s) (p-value = 0.0001). Thus, relative to host RNA, viral RNA is enriched on the positive line. As we were able to detect comparable levels of viral RNA on swabs and positive lines, we next prepared libraries from three samples with N1 C_T_ < 25 from both the swab and positive line (**Fig 2B** and **2C**). Regardless of the sample source, sequenced specimens had 20x genome coverage (20 or more reads per nucleotide) of greater than or equal to 99% (with less than 0.02% covered by 1 or fewer bases), signifying that both the swab and positive line from antigen cards can be used for whole-genome sequencing with no loss in coverage. All specimens were the Alpha variant (PANGO lineage B.1.1.7).

## Discussion

Unlike previous pandemics, global genomic surveillance of SARS-CoV-2 has occurred in near real-time [20]. Medical, DoD, and government institutions actively rely on genomic sequencing data to support public health decisions, underscoring the need for continued sequencing as the virus and pandemic evolve. For institutions that have adopted antigen testing, we demonstrate that it is possible to continue genomic surveillance by sequencing from antigen cards. RNA of sufficient quantity and quality for targeted sequencing of the SARS-CoV-2 genome can be recovered from antigen cards for several hours after completion of the test (**Fig 2**). We found that the quality of genome sequences derived from Ag-test samples is comparable to RNA isolated from NP swabs collected for RT-PCR (**Fig 2**). Furthermore, a comparison of sample sources, antigen card swab vs. lateral flow assay (LFA) positive line, showed that viral RNA from both sources can generate high quality sequencing libraries (**Fig 2**). For ease of use and biosafety concerns, we recommend collecting the swab over the LFA positive line as both specimen types produced comparable libraries.

The present work is a first step for future studies examining the sequencing utility of specimens collected for other antigen or rapid molecular tests. Antigen card specimens present several limitations when compared to NP swabs collected in viral transport media (VTM). First, in the workflow presented here, antigen test swabs are stored in 500 μL of DNA/RNA shield while NP swabs are stored in up to 3 mL of VTM. Multiple RNA extractions can be carried out from VTM specimens after the initial round RT-PCR testing but, antigen swab-derived viral RNA can only be extracted once. Next, variants with novel or interesting mutations can be cultured from VTM lacking inactivating reagents, enabling downstream biochemical or viral neutralization assays. In contrast, antigen tests immediately expose the specimen to an extraction buffer which disrupts the viral membrane, releases viral RNA, and enables the presentation of viral nucleocapsid antigens to anti-nucleocapsid antibodies on the LFA positive line. Thus, viruses collected post antigen-test are most likely non-culturable, however we did not evaluate cultivability. To strengthen the data presented here, studies using a larger number of clinical specimens or evaluating other rapid diagnostic tests should be carried out. Furthermore, institutions that choose to adopt antigen-based whole genome sequence pipelines must work with clinicians and institutional review boards (IRBs) to ensure that clinical specimens are retained for genome surveillance purposes. Clinical researchers must also work with clinicians to ensure proper storage of the antigen card swab prior to transportation to the sequencing laboratory.

To date, our lab has sequenced over 200 excess clinical specimens from antigen cards, thereby supporting local and DoD efforts to track and monitor the emergence of new SARS-CoV-2 variants. This work is a robust demonstration of the viability of sequencing from positive antigen tests and may serve as the groundwork for future studies evaluating additional rapid molecular tests. Sequencing SARS-CoV-2 from antigen cards expands the genomic toolkit available for surveillance and molecular epidemiology activities, which is crucial as the virus evolves and adapts over time.

## Materials and methods

### Specimen acquisition

Specimens were collected under an exempt research protocol approved by the 59^th^ MDW Institutional Review Board (IRB # FHW20200103E) with a waiver of informed consent for the use of residual specimens for sequencing and epidemiological studies. The BinaxNOW™ COVID-19 Ag Card (Abbott) was used to test basic medical trainees for SARS-CoV-2 [17]. Excess clinical specimens were de-identified, the entire card placed in a plastic biohazard bag, and samples transferred to the Clinical Investigations and Research Support (CIRS) laboratory.

### Specimen preparation

The antigen card swabs were gently removed from completed tests, placed in a 15 mL conical tube with 500 μL DNA/RNA Shield (Zymo Research, Catalog #R1100), and stored at 4°C until RNA extraction. The positive line of the LFA test strip was excised and placed in 500 μL of DNA/RNA shield and stored in a 2 mL cryovial at 4°C until RNA extraction.

### RNA extraction

Samples were extracted using the EZ1 Virus Mini Kit v2.0 (Qiagen, Catalog # 955134), per the manual. 400 μL of sample was extracted and eluted into 60 μL AVE buffer (supplied in the kit). The following controls were run with each extraction: (1) positive control, which was a nasopharyngeal swab of SARS-CoV-2 diluted to achieve a C_T_ of approximately 25, and (2) a negative control, which was 200 μL nuclease-free water and 200 μL of DNA/RNA Shield. The extracted RNA was frozen at -80°C or used immediately for RT-PCR.

### RT-PCR

The following primers were utilized: N1 Forward, 5’-GACCCCAAAATCAGCGAAAT-3’, N1 Reverse, 5’-TCTGGTTACTGCCAGTTGAATCTG-3’, N1 FAM probe, 5’-(FAM)ACCCCGCATTACGTTTGGTGGACC(3’-BHQ-1)-3’, RP Forward, 5’-AGATTTGGACCTGCGAGCG-3’, RP Reverse, 5’-GAGCGGCTGTCTCCACAAGT-3’, and RP Cy5 Probe 5’-(Cy5)TTCTGACCTGAAGGCTCTGCGCG(3’-BHQ-3)-3’. 20 μL reactions (15 μL master mix + 5 μL RNA) were prepared using TaqPath™ 1-Step Multiplex Master Mix (No ROX) (ThermoFisher Cat. # A28523) and 20X primer/probe mix. The final primer concentrations per reaction were: N1 Forward & Reverse Primers (400 nM), N1 Probe (200 nM), RF Forward & Reverse Primers (200 nM), and RP Probe (100 nM). The plate was run on the QuantStudio 7 under the following conditions: 25°C for 2 min, 53°C for 10 min, 95°C for 2 min, and 45 cycles of 95°C for 3 sec then 60°C for 30 sec. Fluorescence was detected at the end of each 60°C cycle.

### Library preparation and sequencing

Paragon Genomics’ CleanPlex® FLEX SARS-CoV-2 Panel (Cat. 918014) for Illumina platforms was used to prepare sequencing libraries (starting concentration of 10–50 ng RNA per sample). As a positive control, sequencing libraries were also prepped for VR-1986D, Genomic RNA from SARS-Related Coronavirus 2, Isolate USA-WA1/2020. Library quality and concentration was assessed via fragment analysis using Advanced Analytics’ High Sensitivity NGS Fragment Analysis Kit (Cat. DNF-474-0500). Library quality ratio scores (QRS) were determined by dividing the fragment analysis 250–350 bp peak concentration (ng/μL) by 50–190 bp peak concentration (ng/μL): excellent (QRS >10), Good (QRS 1.0–10), Fair (QRS <1 and >0.5), Poor (QRS < 0.5). Libraries with QRS > 1.0 were denatured and diluted to a final loading concentration of 10 pM following the Illumina MiSeq System Denature and Dilute Libraries Guide (Document # 15039740 v10), and then sequenced on the MiSeq system at 2 x 151 bp using the MiSeq v3, (600 cycle) kit (Illumina, Cat. MS-102-3003).

### Bioinformatics

Illumina adaptor sequences were trimmed using the BaseSpace Onsite FASTQ Toolkit v1.0.0. Adapter trimmed FASTQ files were aligned to the SARS-CoV-2 reference genome (NC_045512.2) using Illumina’s DRAGEN Bio-IT Platform. Primer sequences were removed using the Linux environment fgbio toolkit (bcftools) and a tab delimited file with primer genomic coordinates provided by Paragon Genomics. The DRAGEN was used to create variant call files from primer trimmed BAM files and consensus FASTA files were created using the fgbio toolkit. Genome coverage uniformity and mapping was visualized in IGV (BAM and VCF files). Because the primary goal of this work is to support DoD public health surveillance, the viral sequences generated were not released to patients’ health records but were instead provided to local DoD public health officials for necessary interventions. Concurrently, data were reported to the Armed Forces Health Surveillance Division (AFHSD), FASTQ files uploaded to the NCBI Sequence Read Archive BioProject, and FASTA sequences uploaded to the GISAID EpiFlu Database [21]. FASTA sequences were uploaded to the GISAID EpiFlu Database under the following accession numbers: EPI_ISL_6473528; EPI_ISL_6474667; EPI_ISL_6475107; EPI_ISL_6475110. FASTQ files are published under SRA BioProject accession # PRJNA781677.
